# Provider reported barriers and solutions to improve testing among tuberculosis patients ‘eligible for drug susceptibility test’: A qualitative study from programmatic setting in India

**DOI:** 10.1371/journal.pone.0196162

**Published:** 2018-04-20

**Authors:** Hemant Deepak Shewade, Arun M. Kokane, Akash Ranjan Singh, Malik Parmar, Manoj Verma, Prabha Desikan, Sheeba Naz Khan, Ajay M. V. Kumar

**Affiliations:** 1 International Union Against Tuberculosis and Lung Disease (The Union), New Delhi, India; 2 International Union Against Tuberculosis and Lung Disease (The Union), Paris, France; 3 Department of Community Medicine and Family Medicine, All India Institute of Medical Sciences (AIIMS), Bhopal, Madhya Pradesh, India; 4 World Health Organization, Country Office in India, New Delhi, India; 5 State TB cell, Department of Health and Family Welfare, Bhopal, Madhya Pradesh, India; 6 Bhopal Memorial Hospital and Research Center, Bhopal, Madhya Pradesh, India; Instituto de Diagnostico y Referencia Epidemiologicos, MEXICO

## Abstract

**Background:**

In a study conducted in Bhopal district (a setting with facility for molecular drug susceptibility testing (DST)) located in central India in 2014–15, we found high levels of pre-diagnosis attrition among patients with presumptive multi drug-resistant tuberculosis (MDR-TB)–meaning TB patients who were eligible for DST, were not being tested.

**Objectives:**

In this study, we explored the health care provider perspectives into barriers and suggested solutions for improving DST.

**Methods:**

This was a descriptive qualitative study. One to one interviews (n = 10) and focus group discussions (n = 2) with experienced key informants involved in programmatic management of DR-TB were conducted in April 2017. Manual descriptive thematic analysis was performed.

**Results:**

The key barriers reported were a) lack of or delay in identification of patients eligible for DST because of using treatment register as the source for identifying patients b) lack of assured specimen transport after patient identification and c) lack of tracking. Extra pulmonary TB patients were not getting identified as eligible for DST. Solutions suggested by the health care providers were i) generation of unique identifier at identification in designated microscopy center (DMC), immediate intimation of unique identifier to district and regular monitoring by senior TB laboratory and senior treatment supervisors of patients eligible for DST that were missed; ii) documentation of unique identifier at each step of cascade; iii) use of human carriers/couriers to transport specimen from DMCs especially in rural areas; and iv) routine entry of all presumptive extra-pulmonary TB specimen, as far as possible, in DMC laboratory register.

**Conclusion:**

Lack of assured specimen transport and lack of accountability for tracking patient after identification and referral were the key barriers. The identification of patients eligible for DST among microbiologically confirmed TB at the time of diagnosis and among clinically confirmed TB at the time of treatment initiation is the key. Use of unique identifier at identification and its use to ensure cohort wise tracking has to be complemented with specimen transport support and prompt feedback to the DMC. The study has implications to improve detection of MDR-TB among diagnosed/notified TB patients.

## Introduction

Globally, tuberculosis (TB) is a major public health problem and the increase in multidrug or rifampicin-resistant tuberculosis (MDR/RR-TB) is a growing concern. India has disproportionately high burden of both TB (27% of global burden) and MDR-TB (24% of global burden), relative to population [1]. MDR-TB is defined as resistance to both Isoniazid and Rifampicin with or without resistance to other first line anti-TB drugs [1].

Globally in 2016, of the estimated 600 000 MDR/RR-TB patients, 153 119 (26%) were reported to be diagnosed and 129 689 (22%) were initiated on treatment: indicating majority of MDR-TB patients are lost in the cascade before diagnosis. India, Indonesia and Nigeria alone accounted for almost half of this gap [1]. Studies worldwide have reported high rates of pre-diagnosis attrition in the MDR-TB care cascade including in settings providing molecular drug susceptibility testing (DST) [2–10].

The situation in India mirrors the global picture. [11]. In 2016, of an estimated 147,000 MDR/RR-TB patients in India, only 33, 820 patients were detected giving a case detection rate of 23% and a total of 32, 682 (22%) were put on treatment. [12,13]

Keeping in mind the available resources for MDR-TB diagnosis, India’s revised national tuberculosis control programme (RNTCP) during 2012–17 identified certain TB patients as ‘high risk’ for MDR-TB (referred to as presumptive MDR-TB or TB patients eligible for DST) and were prioritized for DST [14]. RNTCP had limited cohort-wise information on whether all these patients were identified and underwent diagnostic assessment and whether all those diagnosed as MDR-TB were initiated on treatment according to the programme guidelines [15].

**Fig 1:**
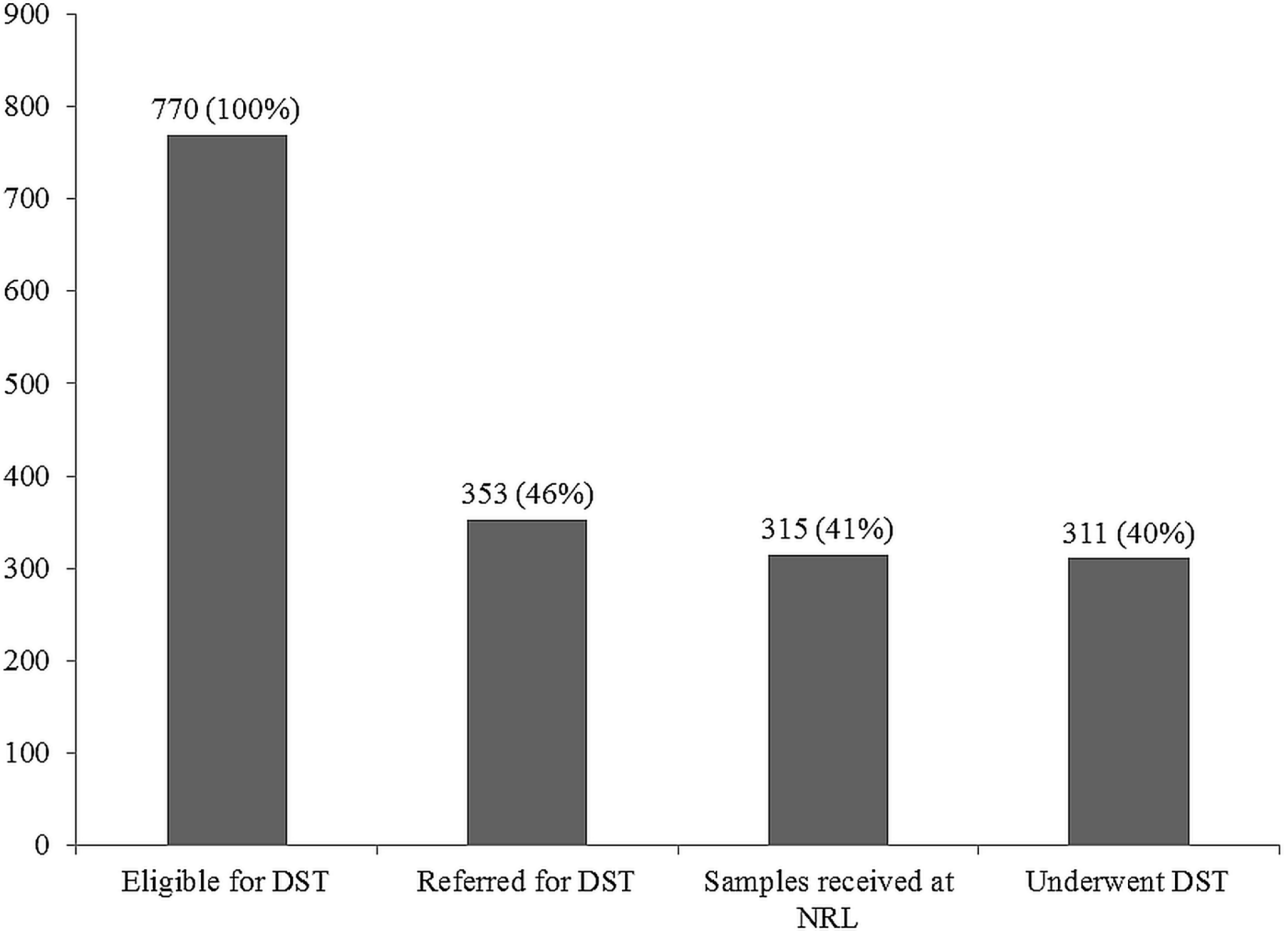
Attrition of patients with presumptive MDR-TB (eligible for DST) in the diagnosis pathway, district Bhopal, India (2014)* [16]. MDR-TB: Multi drug-resistant tuberculosis, DST: Drug susceptibility testing, NRL: national reference laboratory *reproduced with permission, original publisher BioMed Central [16].

A cohort study of care cascade conducted in programmatic settings of Bhopal district (a district in Central India having facility for molecular DST) revealed that pre-diagnosis attrition was 60% among presumptive MDR-TB **(Fig 1),** while pre-treatment attrition was 13% among confirmed MDR-TB (2014). The turnaround times were satisfactory for those who completed the care pathway. Most of the pre-diagnosis attrition was contributed by failure to refer eligible patients for molecular DST by programme staff [16,17]. Despite being in contact and engaged with the TB programme (these ‘high risk’ patients were already diagnosed or registered for treatment within the programme), all eligible TB patients did not undergo DST as per guidelines. This is a major ‘lost’ opportunity in identifying MDR-TB.

There are few qualitative studies on barriers for pre-diagnosis attrition among MDR-TB [9,18–20] and to the best of our knowledge, none of the studies reported solutions to address the barriers. As per the national strategic plan to eliminate TB (2017–25), India is gearing up to provide universal DST–establish a DST facility in every district and offer DST to every notified TB patient at diagnosis in a phased manner [15,21]. So, understanding the barriers and solutions for improving DST from a programme perspective is highly relevant.

Hence, this study was conducted to explore from the health care provider perspective, the barriers and suggested solutions for improving DST in programmatic setting in Bhopal district, India.

## Methods

### Study design

This was a descriptive qualitative study. The theoretical framework underpinning this study was content analysis [22].

### Study setting

#### General setting

Bhopal is a predominantly urban district (population 2.53 million) situated in the state of Madhya Pradesh. RNTCP infrastructure includes one District TB Center (DTC), five sub-district level programme management units (Tuberculosis Units—TU) and 24 designated microscopic centers (DMCs) for sputum smear microscopy. Berasia TU is the only TU that is predominantly rural. Among 24 DMCs, six are located in medical colleges, five in district level hospitals and 13 in primary/secondary level health centers. Details of sputum smear microscopy at diagnosis and follow up are entered in laboratory register at DMC. Details of patients registered for treatment in programme setting (public) were entered in TB treatment register at TU level. Since early 2017, it is recommended that all diagnosed TB patients (both in the public and the private sector) are notified to NIKSHAY (a web-based, case-based reporting system under RNTCP) and entered in TB notification register kept at each public health facility [23].

#### Programmatic management of DR-TB (PMDT) services

Patients eligible for DST in 2014–15 included all ‘previously treated’ patients, any patient who was smear positive during follow-up sputum microscopy, patients with pulmonary TB who were contacts of known MDR-TB and all HIV-TB co-infected patients at diagnosis [14]. Since 2016, the programme also took a policy decision to offer upfront molecular DST (cartridge-based nucleic acid amplification test-CBNAAT) to diagnose extra pulmonary TB, paediatric TB and TB among people living with HIV. Case definitions used in this study have been described in **Table 1**[24].

**Table 1:** Case definitions as per revised national tuberculosis control programme (RNTCP), India [24].

**New patient**		A patient with TB who has never had treatment for TB or has taken anti-TB drugs for less than one month
**Previously treated patients**		Received one month or more of anti-TB drugs in the past
	**Recurrent TB**	A patient with TB previously treated and declared as successfully treated (cured / treatment completed) and is subsequently found to be microbiologically confirmed TB patient
	**Treatment after failure**	Previously treated and whose treatment failed at the end of their most recent course of treatment
	**Treatment after loss to follow up**	A previously treated patient and was declared loss to follow up in their most recent course of treatment and subsequently found microbiologically confirmed TB patient
	**Others**	A previously treated patient with TB but whose outcome after their most recent course of treatment is unknown or undocumented. (This subgroup mostly refers to previously treated patients who are smear negative)
**Follow-up smear-positives**		A patient with TB whose follow up sputum is positive during any of the routine follow up
**HIV associated with TB**		A patient with TB who is a previous known patient of HIV or gets diagnosed as HIV during diagnosis of TB or anytime during TB treatment

TB–Tuberculosis; HIV–Human immunodeficiency virus

In Bhopal district, the diagnostic facility (National Reference Laboratory—NRL) is located in a district level, tertiary, public health care facility, named ‘Bhopal Memorial Hospital and Research Center’. The diagnostic facility is certified by the RNTCP for phenotypic (solid/liquid culture and DST) and molecular DST (line probe assay—LPA and CBNAAT). In January-March 2014, if sample was smear positive, then LPA was used upfront. Among smear negative samples, culture was done followed by LPA, if culture turned out to be positive. From April 2014 onwards, LPA was used for smear positive and CBNAAT was used for smear negative samples (CBNAAT was introduced in NRL in April 2014) [16]. Since February 2016, an intermediate reference laboratory (IRL) has been started in the TB hospital of the district which also provides CBNAAT services.

Treatment for MDR-TB was provided at DR-TB center at TB hospital, Bhopal according to national guidelines which were in the line with WHO recommendations [14]. Patients with RR-TB were also treated with the standardized MDR-TB regimen. Therefore, in the study, MDR-TB included RR-TB as well.

#### Care pathway

**Fig 2:**
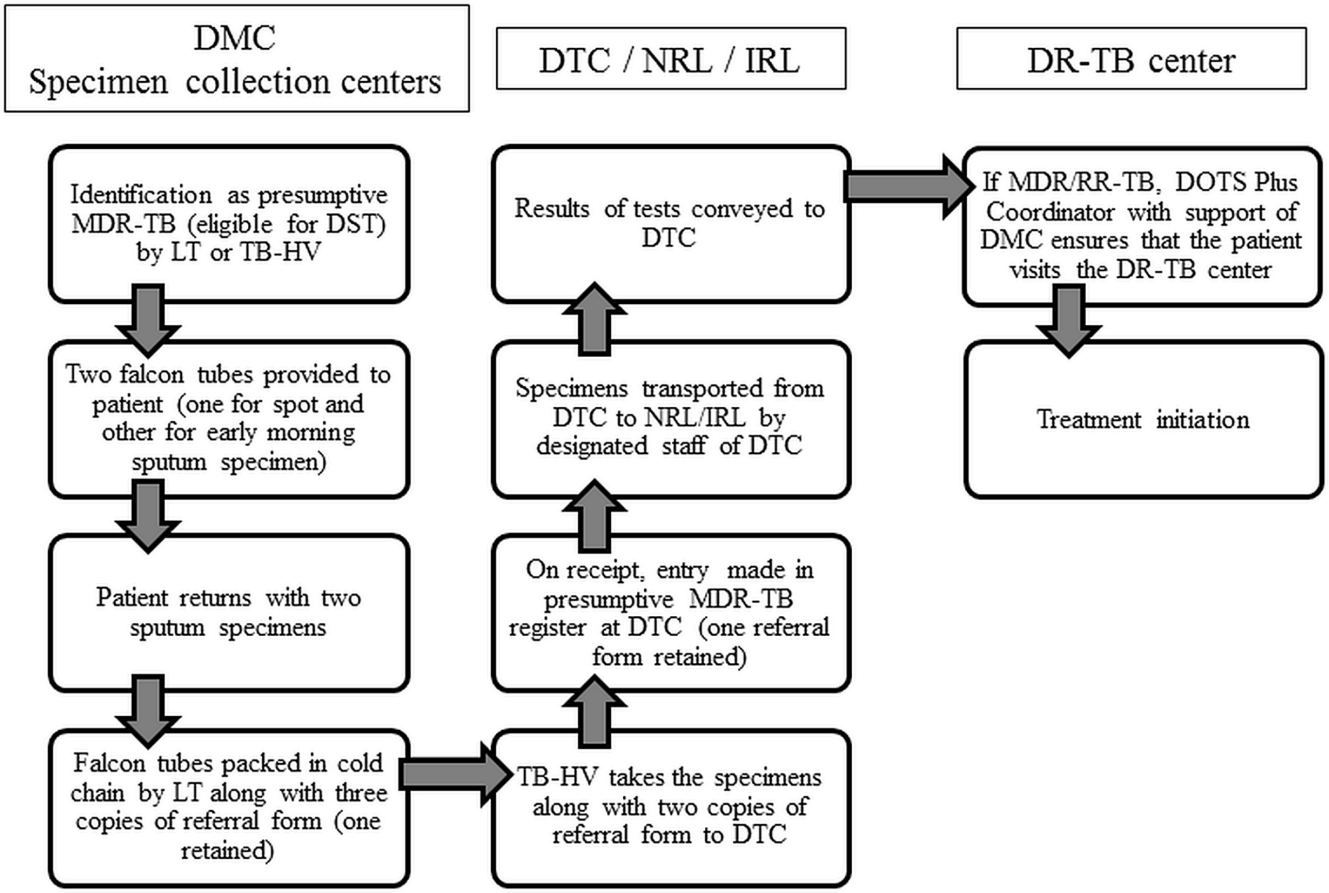
Cascade of care of patients with presumptive MDR-TB (eligible for DST) in the diagnosis pathway, district Bhopal, India*. TB–Tuberculosis; DST–drug susceptibility testing; MDR-TB–multi drug-resistant tuberculosis; DMC–designated microscopy center; DTC–district tuberculosis center; DOTS–directly observed treatment short course; NRL–national reference laboratory; IRL–Intermediate reference laboratory; DR-TB–drug-resistant TB; LT–laboratory technician; TB-HV–TB health visitor.

The DMC laboratory technicians were expected to identify those eligible for DST among bacteriological confirmed patients at diagnosis/follow up. The TB health visitors were expected to use treatment register to identify those eligible for DST among clinically confirmed patients at treatment initiation. The care pathway for diagnosis of MDR-TB is shown in **Fig 2.**

### Study population

Health care providers involved in PMDT in Bhopal district (April 2017) were the study population. They were one district TB officer (head of the TB programme in the district), one senior DR-TB/TB-HIV supervisor (the paramedic who coordinates with MDR-TB patients and treatment centres for the treatment initiation and follow-up), six laboratory technicians (LT—the paramedic responsible for sputum microscopy at DMC, which caters to a population of ~0.1 million), one senior TB laboratory supervisor (STLS—the paramedic responsible for monitoring the DMCs in an area of 0.5 million population), two TB health visitors (TB-HV—the paramedic responsible for treatment support and follow up of TB patients for a population of 0.1 million), two senior treatment supervisors (STS—the paramedic responsible for supportive supervision, recording and reporting TB treatment for a population of 0.5 million), eight directly observed treatment (DOT) providers (community-based volunteers involved in DOT) and two microbiologists (one each from NRL and IRL).

They were selected after brainstorming among research team members (HDS, AMK, ARS). We purposively (purposive sampling-maximum variation) selected staff who were experienced, vocal and were perceived to provide insights into barriers and suggest solutions for addressing pre-diagnosis attrition.

### Data collection

Data collection was done in April 2017. Barriers and suggested solutions to improve testing were explored through one to one interview and focus group discussion (FGD) with experienced key informants involved in PMDT implementation. In total, ten interviews and two FGDs were conducted. We used the later interviews/FGDs to dig deeper into what emerged or clarify conflicting ideas from earlier interviews/FGD. At the end after attaining data saturation, an FGD with LTs of DMCs was conducted. As the LTs played a key role before referral and this was also a key gap in the care cascade **(Fig 1)**[16], we explored the perceived feasibility of the suggested solutions with the LTs.

HDS or ARS conducted the interviews/FGDs with the other person taking verbatim notes. HDS and ARS (medical doctors (males) with MD in community medicine and working as senior operational research fellow and senior resident doctor respectively), with post MD experience of five years and two years respectively, were trained and experienced in conducting qualitative research. HDS and ARS were not programme staff. Both were part of the quantitative study that preceded this study [16,17]. ARS had prior interaction with the staff as he and his institution worked in close collaboration with the TB programme in the district.

Data collection was done after obtaining permission and written informed consent to participate in the study. No incentives were given to participate in the study. Participants were approached over phone. Interviews/FGDs were done at the date, time and place convenient to the participants (at their workplaces). Only the participant(s) and the researcher(s) were present during the interview/FGD. Participants were informed about the findings of the previous study in 2014 using bar diagram **(Fig 1)** and explained the purpose of the current study [16]. An interview/FGD guide with broad open ended questions and probes was used after pilot testing **(S1 Annex).** Pilot testing was done by ARS in Bhopal a month before data collection. Questions specific to the role of the health staff were also asked. Audio recording (after consent) and verbatim notes were taken during the interview. Interviews were conducted in a mix of Hindi and/or English. The duration of the interviews were noted. No repeat interviews were carried out. Drop outs, if any, (along with reasons) were noted. After the interview/FGD was over, the summary of the interviews was read back to the participants to ensure participant validation. Field notes (if any) from observations during data collection were made.

### Data analysis

Translation and transcription (in English) of all the interviews/FGDs were made within two months based on the audio records and verbatim notes. ARS and HDS read the transcripts to become familiar with the data. Manual descriptive thematic analysis was used by ARS and HDS to analyse the transcripts [25–27]. It was reviewed by AMK, AMVK and MP to reduce bias and increase interpretive credibility. The decision on coding rules and theme generation were done *a priori* by using standard procedures and in consensus [28]. Any differences were resolved by discussion [25]. To ensure that the results were a reflection of the data, the codes/themes were related back to the original data [29]. The final results arising after qualitative data analysis were shared with the stakeholders for their feedback and approval.

Themes/categories have been reported below in single quotation marks, verbatim quotes in double quotation marks and italicised, author explanation within quotes in square brackets and respondents’ details in round brackets. The findings were reported by using ‘Consolidated Criteria for Reporting Qualitative Research (COREQ)’ [30].

### Ethics

Ethics approval was obtained from the Ethics Advisory Group of The Union, Paris, France (EAG number 64/16) and Institute Human Ethics Committee, All India Institute of Medical Sciences (AIIMS) Bhopal, India (IHEC-LOP/2017/IM0070). Permission and support was sought from the RNTCP programme managers and other relevant authorities. Written informed consent was taken by HDS/ARS before the beginning of interview/FGD. The consent process was approved by the ethics committees.

## Results

**Table 2** depicts the study participant characteristics and duration of each one to one interview / FGD. One laboratory technician from rural area did not provide consent for the one to one interview, because the staff had to leave early to attend to a personal commitment.

**Table 2:** Study participants (health care providers involved in programmatic management of drug-resistant TB) characteristics and duration of each data collection activity in Bhopal district, India (2017).

Method of data collection	Participants’ position	Demographic details	Experience in that position (in years)	Duration of interview / discussion in minutes
One to one interviews (n = 10)				
	DMC laboratory technician	28 years, male	5	30
	Senior TB laboratory supervisor	46 years, male	13	30
	TB health visitor (n = 2)	33 years, male	10	16
		43 years, male	14	38
	Senior treatment supervisor (n = 2)	40 years, male	8	45
		39 years, male	12	25
	District TB Officer	57 years, male	7	35
	Senior DR-TB supervisor	43 years, female	5	40
	Microbiologist (IRL)	34 years, female	2	36
	Microbiologist (NRL)	52 years, female	20	35
Focus group discussion (n = 2)				
	DOT providers (n = 8)	30 years, male	5	45
		27 years, male	3	
		45 years, male	6	
		45 years, male	4	
		46 year, male	8–10	
		25 years, female	2	
		50years, female	7–8	
		37years, female	7–8	
	DMC laboratory technician (n = 5)	49 years, male	10	40
		52 years, female	10	
		51 years, male	9	
		45 years, male	19	
		45 years, male	19	

RNTCP–revised national tuberculosis control programme; DMC–designated microscopy center; TB—Tuberculosis; DR-TB–drug resistant TB; IRL–intermediate reference laboratory; NRL–national reference laboratory; DOT–directly observed treatment

In the background of the care pathway **(Fig 2)**, we present the results in two separate parts: perceived barriers and then suggested solutions.

### Perceived barriers

**Fig 3:**
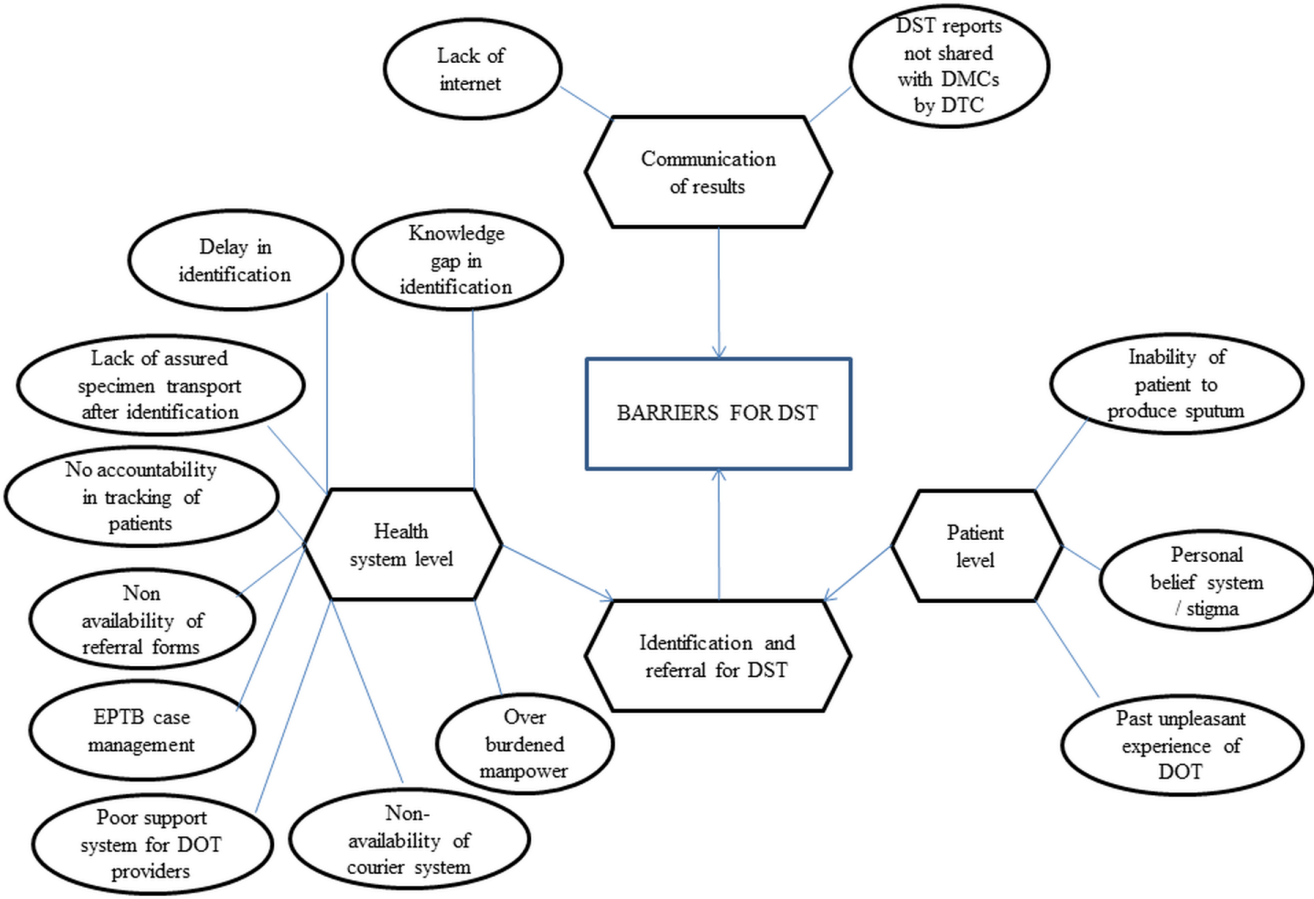
Thematic analysis showing “barriers to undergoing DST among patients with presumptive MDR-TB (eligible for DST)” under programme setting in Bhopal district, India (2017). DST–drug susceptibility testing; MDR-TB–multi drug-resistant tuberculosis; DMC–designated microscopy center; DTC–district tuberculosis center; DOT–directly observed treatment.

We constructed a thematic network organizing the themes around the global theme ‘barriers to DST’ and represented it as a web-like, non-hierarchical figure **(Fig 3).** We identified 14 themes and broadly grouped them into two categories: ‘identification and referral for DST’ and ‘communication of results’. Under the former, two sub-categories were identified: health system level and patient level. We present the perceived barriers below with each heading representing a sub-category/category.

#### Identification and referral for DST–health system level barriers

Nine out of 14 themes were categorized here. To begin with, a ‘knowledge gap in identification’ was detected in few but this did not appear to be consistently seen among many staff.

*“Have heard it* [providing molecular DST to extra pulmonary TB and children with TB] *but do not exactly remember what it means”* (28 year old male staff)

The more common issue was ‘delay in identification’. In such instances, sputum smear positive patients (bacteriologically confirmed) were identified by TB-HV during treatment initiation (as eligible for DST) or when TB treatment register was reviewed to create the line list.

*“Our line list is from TB register to generate eligible* [for DST]. *And check how many have not done* [undergone DST]*”* (40 year male staff)

Once a patient was identified as eligible for DST, the major theme that emerged consistently was ‘lack of assured sputum specimen transportation’. The issue here was of coordinating the patient’s return (to DMC) with two sputum specimens and availability of the TB-HV (to transport the specimens) on the same day at the DMC. Patients were asked to come on a fixed day of the week, which may not have been convenient to them.

*“Sometime patients come but TB-HV cannot go* [take the specimen for DST] *because of unexpected work*. *Sometime we are available but patient does not come”* (40 year old male staff)

*“It appeared that a particular day was fixed for a particular DMC*. *This process of fixing particular day might account for loss of few patients/specimens”* (34 year old female staff)

This problem was exacerbated in rural DMCs which were approximately 30–45 kilometres from the DTC. The TB-HV took the support of village-level DOT providers residing in patient’s village to coordinate patient’s return to DMC with two specimens on the assigned day. The DOT providers faced challenges coordinating patient’s return.

*“Once they go to village it is difficult for them to come back*. *Some roads are not concrete*. *During rains it becomes difficult… it may take almost a day for them to come give specimen and go back*.” (40 year male staff)

The DOT providers also cited ‘lack of support’ from staff at DMC especially in motivating patients to return to DMC and provide sputum specimens.

*“We* [DOT providers] *meet them* [patients eligible for DST] *daily*. *If someone from Bhopal or Berasia* [TU in rural area] *come and motivate them*, *they value it more*.*”* (a DOT provider (male, 45 years) from rural area)

In urban areas, we found instances where patients or patients’ relatives carried the specimen to DTC.

*“All specimens are being sent through patients in our site”* (a 51 year old male staff from urban area)

During 2014, ‘non-availability of referral forms’ was also identified as a theme. This improved in 2015 as the referral forms were available and it was made mandatory to send specimens with referral forms to DTC and keep a copy of the form at the DMC.

Another major health system level theme that emerged was ‘lack of accountability in tracking of patients’. As mentioned above, there were instances where referral form was not used. If referral form was used, the staff did not fill key patient related information which could have been helpful in tracking a patient.

*“Sometimes LT may not* [be able to] *track an eligible patient if he or she doesn't return for providing DST specimens”* (46 year old male staff)

*“It* [those specimens, sent from DMC by LT but say not received at NRL] *does not get tracked*.*”* (46 year old male staff)

The health care staff perceived that they were ‘overburdened’ and this could be one of the reasons for attrition.

*“I am the only staff taking care of laboratory work related to TB*, *HIV*, *ANC* [antenatal care] *and other lab* [laboratory] *services and also the role of TB-HV*. *Hence it may become difficult for me to go* [every day to take the specimen for DST]*”* (a male staff)

*“Sometimes our work load will be at peak especially during winters and rainy seasons*. *We could not even get time to drink water”* (a male LT)

*“They [programme] have started cutting down workers at many levels*. *More workers have to be deployed to peripheral level”* (52 year old female staff)

In rural areas, the programme tried the idea of using a courier system to transport the sputum specimen to NRL/IRL, but that did not succeed. We tried to explore the reasons for this in detail but did not get any relevant information.

*“I* [health staff] *explained to them* [local courier service providers] *that you will get reimbursed in time*. *But still they refused*. *They may know best why they refused”* (40 year old male staff)

‘Extra pulmonary patient management’ was identified as another major theme. Extra-pulmonary TB patients were usually diagnosed and managed at tertiary care facilities. There were challenges in collecting specimens from the extrapulmonary sites. The DMCs in these facilities did not enter details of the extra-pulmonary TB specimens (if specimen collected) in the laboratory register of DMCs. This prevented tracking of the specimen. The staff felt that even if the entry of these specimens was made in the laboratory register, result column would remain blank in most of the instances as the DMC would not be performing any tests on the specimen. Hence, they did not make the entries for extra pulmonary specimens.

*“Even if we register*, *we could not get results of those extra pulmonary specimens*. *So the result column will remain blank most of the times*. (staff working in urban area)

*“RNTCP staff cannot take specimens from extra pulmonary TB patients*. *Many a time even pulmonary TB patients could not produce sputum*. *Broncho alveolar lavage should be done in those instances*. *RNTCP staffs need to be sensitised in these issues*. *Gynaecology*, *orthopaedics and other departments* [in teaching hospitals and other tertiary hospitals] *need to send specimen for DST”* (53 year male staff)

#### Identification and referral for DST–patient level barriers

Patient’s return to DMC with two specimens on the assigned day was also influenced by some patient level barriers. The health care providers perceived that ‘inability to produce sputum’ after a certain period of TB treatment could be a reason why patient did not return with two specimens. ‘Patients’ belief system’ about the TB disease and ‘associated stigma’ were identified and it had its effect not only on ongoing drug susceptible TB treatment but also on undergoing DST. Below are some quotes from DOT providers.

*“Patients used to tell us—I am afraid*. *What if it comes positive?”*

*“They* [presumptive MDR-TB] *do not want to take treatment nor do they want to go to DMC*.*”*

*“If someone* [TB patient] *does not want to understand* [need for DST among TB patient] *what can we do? Do we hit our head against stone? It is difficult to explain presumptive MDR-TB to them* [patients with TB].*”*

*“Once a patient has TB*, *they hide it so much that they come in the dark to get treatment”*

Health care staff also perceived that some patients’ ‘past unpleasant experience of DOT’ could also be a reason for non-cooperation to return to DMC with two sputum specimens.

#### Communication of result

Providers reported lack of internet connection at IRL and having had to use their personal mobile internet. They said that, despite sending the emails (containing the test results) to concerned nodal person at DTC, the ‘reports were not being shared with DMCs’.

*“Initially we used our personal internet connection”* (female staff)

*“We* [IRL/NRL] *used to get call to enquire about the reports* [despite sending reports over email]. *Coordination has to be improved to provide better services to patients”* (female staff)

### Suggested solutions

The suggested solutions were identified as 10 themes, grouped into three categories **(Table 3)**. We present the suggested solutions below with each heading representing a category.

**Table 3:** Suggested solutions as perceived by programme staff to increase drug susceptibility testing among patients with presumptive MDR-TB in Bhopal district, India (2017).

Categories	Themes	Verbatim Quote
Improved patient tracking	Generation of unique identifier	*“This is important and it should be done at the day one itself without any delay*. *There should be some web based mechanisms so that we can track patients easily*.*”* (57 year male staff)
		*We can use adhaar card number* [Unique IDENTIFIER given by Government of India], *now it is mandatory to mention in Nikshay portal also* [Web portal of RNTCP].” (28 year old male staff)
		*“DMC name*, *serial number and year can be used to generate unique id* [identifier]”(40 year male staff)
		*“There could be no problems* [with reference to unique identifier], *we send specimen from here and they may ask us* [tracking] *that specimen not reached”* (28 year male staff)
		*This idea of line listing using unique id is very good*. *LT* [laboratory technician]*can ensure it is done”* (33 year male staff)
		*“We have been given a sim from department to contact each other*.*”* (46 year old male staff)
		*“If it is done*, *it will be great*. *Although it will slightly increase our work load but will help us in ensuring that all* [eligible for DST] *are getting tested also an independent assessment can be done”* (46 year male staff)
		*“I feel that we should get some written acknowledgment for patients whom reached DTC and got tested for MDR* (laboratory technician in urban area)
		*“That will be better*. *LT will identify patients & TB HV will be there along with LT*, *so he can send SMS*. *But these things are individualised*. *Some LTs might be having many works while some may not*. *This intervention may appear feasible to us at programme level*. *But it’s difficult to apply practically”(*34 year old female staff)
	Filling of unique ID in DMC, DTC & IRL / NRL registers and referral forms	*“I am convinced that they should be mentioned* [Unique identifier] *in referral forms*.” (43 year female staff)
	Timely monitoring of line list by STLS to identify missed patients	*“During our routine visit*, *we can do this* [monitoring of line list] *by checking the laboratory register to see if any presumptive MDR has been missed*.” (46 year male staff)
		*“May be weekly line listing has to be done in better way*. *We can do daily monitoring of laboratory personnel*, *once the practice is established we can shift to weekly monitoring*. *We should also check the referral register*.*”* (34 year female staff)
Improve specimen transport support mechanism	NGO support	*“If NGO* [non-Governmental organization] *can get specimen from home to DMC it will be very helpful*. *This is rural area*, *hence specimen collection from patient’s home will help*. *Days I will coordinate*.*”* (33 year male staff)
		*“If we adapt a responsible NGO for transporting specimens*, *it will be of great help for those LTs* [laboratory technicians] *working in rural areas*.*”* (laboratory technician in urban area)
	Reimbursement for transport to programme staff	*“If we* [DOT providers] *get such facilities* [monetary incentives] *if we get some benefit why won't we work*. *We worked a lot under those* [other projects run by non-Governmental organizations] *projects*. *Even if we do something good*, *we get their blessings*” (DOT provider from Rural area)
		*“Incentives [reimbursements] should be given for the health workers*. *When he doesn’t have any out of pocket expenditure*, *he will be more motivated to work”* (43 year male staff)
Awareness/training to other stakeholders	Awareness among patients	*“Without creating awareness among patients*, *we can’t improve the situation*. *Creating awareness among patients is again a challenging job*.*”* (43 year male staff)
	Training of private practitioners	*“We are not conducting training sessions regularly* [for private practitioners] *but*, *I am aware that few such sessions had been organised in our district* [To communicate with medical officers regarding criteria for PMDT].*”* (43 year male staff)
	Training to Medical college & programme staff	*“There is a need for training of general health system doctors and other staffs*. *Maybe they're interested in diseases where patient load is huge*.*”* (43 year old female staff)
		*“Programme keeps on changing*. *If we need good results*, *not only senior DR-TB TB-HIV supervisor*, *all workers have to be trained*. *Training should be extended to non RNTCP staffs too”* (57 year male staff)
		*“I am not sure whether medical college hospital staffs had undergone hands on training*. *But*, *they need to be trained”* (57 year male staff)

Verbatim quotes in double quotation marks and italicised, author explanation within quotes in square brackets and respondents’ details in round brackets.

### Improve patient tracking

There were three themes identified under this category: ‘generation of unique identifier’, ‘filling of unique identifier in DMC, DTC and IRL/NRL register and referral form’ and ‘timely monitoring of DMC line list by STLS’.

A unique identifier consisting of laboratory code, laboratory serial number and year could be generated and immediately shared by the LT with a nodal point in DTC (either through short messaging service or using internet) and entered in the presumptive MDR-TB register at DTC. There were other suggestions for unique identifiers as well which were not found to be intuitive. When the patient is referred or specimen is transported, this unique identifier should be entered in referral form. The unique identifier should also be entered at the DTC presumptive MDR-TB register and IRL/NRL laboratory register once the specimen is received. The STLS and STS during routine supervisory visits could cross check the laboratory register and the TB register respectively and identify missed patients that were not included in the presumptive MDR-TB line list.

### Improve specimen transport support mechanism

Improved patient tracking should be supported with improved specimen transport mechanisms. Providers opined that this is possible through the ‘support of local NGOs’ especially in rural areas and provision of ‘reimbursement to programme staff’.

### Awareness / Training to other stakeholders

Generating awareness among patients, private practitioners and training medical college and programme staff especially regarding extra pulmonary specimens was suggested. If entry of extra pulmonary specimens is made possible in the laboratory registers, then it was felt that the above suggested tracking mechanism will cover them.

**Fig 4:**
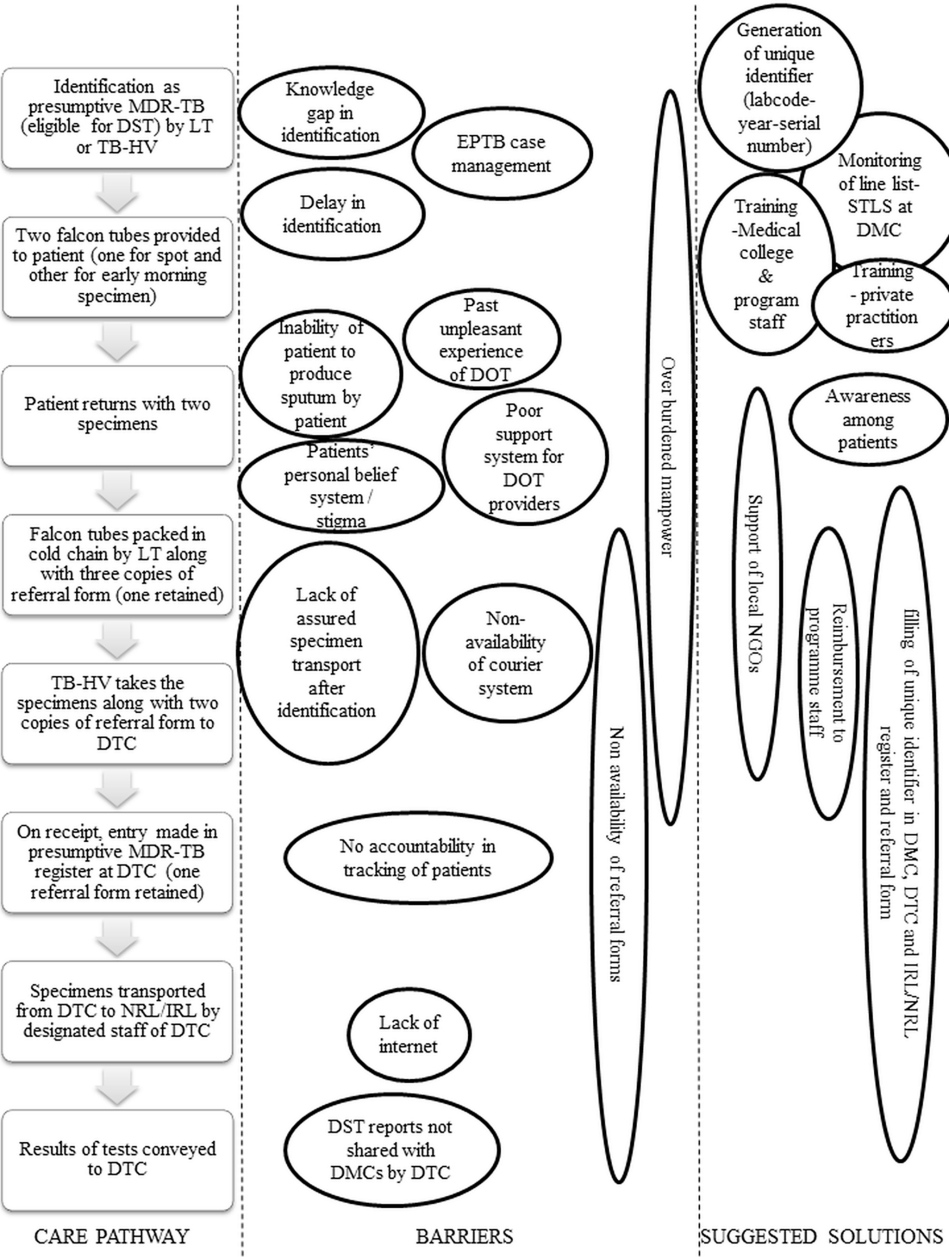
Providers’ perspectives into barriers and suggested solutions to improve DST among presumptive MDR-TB (eligible for DST) in Bhopal district, India (2017), in relation to the care pathway for diagnosis*. TB–Tuberculosis; DST–drug susceptibility testing; MDR-TB–multi drug-resistant tuberculosis; DMC–designated microscopy center (sputum collection center); DTC–district tuberculosis center; DOTS–directly observed treatment short course; NRL–national reference laboratory; IRL–Intermediate reference laboratory; DR-TB–drug-resistant TB; LT–laboratory technician of DMC; TB-HV–TB health visitor; NGO–non-Governmental organization; STLS–senior tuberculosis laboratory supervisor.

Providers’ perspectives into barriers and suggested solutions to improve DST among eligible patients, in relation to the care pathway for diagnosis, have been visualized in **Fig 4**.

The LTs generally thought that the suggested solutions could be a feasible strategy, however, they had some apprehensions as increase in denominator of patients eligible for DST and possible non-redressal of specimen transport related issues could result in poorer performance indicators at their level (proportion of eligible undergoing DST). They were in support of this strategy subject to improvement of specimen transport mechanisms. **(Table 3)**

## Discussion

### Interpretation of perceived barriers

We identified lack of assured transport of specimen and lack of tracking after identification and referral as the main reasons for the pre-diagnosis attrition among MDR-TB patients. There were many health-system related issues preventing specimen collection from patients and transportation to the laboratory. First the patient had to return to DMC to submit specimens for DST on a particular day only. If the patient returned, the LT and TB-HV had to be available in the DMC. The process of reimbursement for TB-HV to carry specimen for DST was in place; despite this, assured specimen transport by TB-HV was not happening especially in rural areas. If available in DMC, the TB-HV must not have any other planned or emergency engagements. Only if the specimen passed all these barriers and reached the DTC, an entry was made in the presumptive MDR-TB register. If the patient’s specimen did not reach, there was no way for the DTC to track it. As a result, the programme missed three categories of patients: a) those that were not identified b) identified, but not referred and c) those who were referred, but the specimens did not reach the DTC.

Issues at the level of patients’ return to DMC for submitting samples were identified. This may be due to poor knowledge, negative expectations, providers’ discriminatory attitudes, patient’s past unpleasant experience of DOT and stigmatization which have also been reported in the past from India [31–33]. In a setting providing CBNAAT in South Africa (2010–12), poor perception of public sector services were common among patients and possibly resulted in deferring health-seeking and patient seeking health care from private sector [18]. In China (2012–13), poor TB knowledge and socioeconomic barriers were the primary reasons for patient level delay[19].

Another possible reason was delay in identification due to the practice of line listing all the eligible patients from the TB treatment register/notification register. Eligible microbiologically confirmed patients could be picked up early from the DMC laboratory register. It may take as long as 30 days for the patient to get registered after diagnosis and treatment initiation. Patients return to their home and many may feel symptomatically better due to one month of treatment. This may be the reason why the patient’s may be unable to produce sputum. The right moment to ensure that DST is done for microbiologically confirmed patients is when the patient is diagnosed. This might have been missed in many instances.

Extrapulmonary specimen obtained from patient with presumptive extrapulmonary TB should be sent for DST for simultaneous diagnosis of TB and MDR/RR-TB [24]. Providers reported that collecting specimens from extrapulmonary sites was a challenge and required special skill and expertise. If collected at tertiary care facilities, the other challenge was poor documentation at DMCs in these facilities. The laboratory register at DMC was used for documenting results of sputum specimens only (up to 2016).

A study was done in Puducherry, south India (2013–14), where some themes common to our study were identified: 'lack of a systematic mechanism to track referrals for culture and drug susceptibility testing', 'absence of courier service to transport sputum' and 'lack of knowledge and ownership among staff of general health system' [9]. In Vietnam, providers revealed insufficient screening capacity at district hospitals, poor communication and implementation of policy changes and inconsistent training as the reasons for the large gap in estimated and diagnosed MDR-TB [20].

### Interpretation of suggested solutions

Based on the suggested solutions, the programme may consider the following. Since 2017, the revised laboratory registers and TB notification registers have been introduced. The DMC laboratory register is more detailed compared to the previous register and collects information on past TB treatment, vulnerable populations, HIV status, option for type of specimen and whether specimen was sent for DST (if applicable). According to the recently updated PMDT guidelines (2017), presumptive TB patients from vulnerable populations (clinical, social and geographically vulnerable) are also eligible from upfront CBNAAT [34]. The STLS during the routine supervisory visits can check on the completeness of the laboratory register. Laboratory register and TB notification register should be used to promptly identify microbiologically confirmed and clinically confirmed eligible patients respectively.

Second, cohort wise tracking of patients eligible for DST should be initiated through the generation (at DMC) and intimation (to DTC) of unique identifier during identification. This unique identifier can be ‘labcode-year-serial number’ or the TB notification number depending on whether patient is identified from DMC laboratory register or TB notification register. The STLS and STS can monitor the DMC laboratory register and TB notification register respectively to ascertain whether any eligible patients have been missed. Third, unique identifier should be recorded at each step of care pathway including NRL/IRL.

Fourth, systems for improved and assured specimen collection and transport from all the DMCs in Bhopal district to DST facility should be initiated especially in rural areas. NGO volunteers can coordinate the visit of the patient to DMC with two sputum specimens and transport the packed specimens to DTC/IRL/NRL. Other potential options are to provide honorarium to village-based DOT providers to play this role or use speed post or courier services. Fifth, for vulnerable populations including extra pulmonary TB, people living with HIV and childhood TB [34], in addition to routine sensitization of staff, all diagnosed patients’ entry should be done in DMC laboratory register and TB notification register in the tertiary care facilities of the district where such patients are managed and molecular diagnostic tests should be done upfront at the time of TB diagnosis itself. Mechanisms to improve the coordination between the programme and the tertiary care hospitals should be looked into. Finally, timely feedback should be provided to the DMC from the DTC regarding the patient whose unique identifier has been intimated.

### Implications for the programme

All national programmes are dynamic. Therefore, the suggestions offered by the programme staff should be modified in line with programmatic changes.

Under NIKSHAY, DMCs are expected to search for TB patient already registered in NIKSHAY or if not already registered, generate a NIKSHAY identifier and upload the request for DST online. However, this did not come out in the qualitative data because the infrastructure for implementing this at DMC level (computers and internet in DMCs) was not in place in Bhopal. Once in place, emphasis on regular monitoring of data entry with dates of DST request and DST in NIKSHAY and use of this data for improving patient tracking need to be considered. The presumptive MDR-TB register at DTC may not be then required as tracking would be possible through NIKSHAY. In addition, NIKSHAY identifier may suffice and replace the unique identifier suggested by the programme staff in this study.

To close in the large gap between estimated MDR-TB and those diagnosed, the practical step is to first detect MDR-TB among all notified TB patients. As India gears for upfront DST for vulnerable populations and universal DST over time, the solutions suggested in this study shall remain applicable at a much larger scale [21,34]. However, there may not be a need to create a line list of patients eligible for DST once universal DST is implemented.

### Strengths

This study has implication not just nationally but internationally as well. This is the first qualitative study globally to provide suggested solutions from provider perspective to improve DST. This is only the second study from India to look at the ‘why’ of pre-diagnosis attrition among patients with presumptive MDR-TB [9]. In Puducherry, India (2013–14), the ‘how’ component (suggested solutions) were not studied and the recommendations in that paper were based on authors’ interpretation [9] The recommendations in this paper have come from the programme staff who were involved in implementation. This is a major strength of this study.

### Limitations

There are some limitations of the study. We did not delve into patients’ perspective. Patient-level factors identified through a programme perspective, as expected, tended to blame the patient. However, it is important to study this in future.

We did not collect data from the medical officers in the general health care delivery system as the LTs and TB-HVs were provided the instructions to identify those eligible for DST at diagnosis and treatment initiation respectively. A presumptive TB patient not getting referred to DMC by the medical officer / doctor is a generic problem that has been identified in other studies [35,36]. Our study did not address the barriers among patients visiting private facilities and patients not visiting any health facility. Perspective of programme managers working at national level could have been considered.

### Future research

Future research should look into feasibility and effectiveness of the suggested solutions after consultation with the programme in possibly decreasing the pre-diagnosis attrition among TB patients eligible for DST. Pragmatic randomised intervention trials may be used to test the effectiveness and impact of interventions to improve follow-up of referrals.

## Conclusion

Limitations notwithstanding, our study makes important contribution to our understanding of barriers for undergoing molecular drug susceptibility testing among eligible TB patients. Lack of assured specimen transport and accountability for tracking patient after identification and referral were the key barriers. The identification of eligible patients among microbiologically confirmed TB at the time of diagnosis and among clinically confirmed TB at the time of treatment initiation is the key. Generation of unique identifier after identification (and immediately sharing it with DTC) followed by use of this unique identifier across the registers at various stages would help in tracking the patients. This has to be complemented with specimen transfer support and constant feedback to the DMC about the identified patient. The study has implications to improve detection of MDR-TB among diagnosed/notified TB patients. However, though the principles remain the same, the suggested solutions by programme staff need to be modified from time to time for them to be in line with the programme.

## Supporting information

S1 Annex Interview / Focus group discussion guide used during data collection, Bhopal, India (2017). (DOCX)
