# Supplementary material for: Provider reported barriers and solutions to improve testing among tuberculosis patients ‘eligible for drug susceptibility test’: A qualitative study from programmatic setting in India
Source: PLoS One. 2018 Apr 20;13(4):e0196162. doi: 10.1371/journal.pone.0196162 (PMC5909888; doi:10.1371/journal.pone.0196162)
Supplement: S1 Annex — (DOCX) [file pone.0196162.s001.docx]

**Key informant Interview / FGD guide**

**Name of the participant (s):**

**Designation:**

**Date of Interview:**

**Interview start time:**

**Interview end time:**

**Name of the Interviewer:**

After a brief introduction to the participant(s) regarding the findings of the study in 2014 (show the figure as a conversation starter) and purpose of the interview, the interviewer will take informed written consent for the interview/FGD. Written informed consent will also be requested for audio recording

1. Programmatic Management of Drug-resistant Tuberculosis (PMDT) has been initiated in Bhopal district. What do you think are the positive points about PMDT services? How does it benefit the patients? [say CbNAAT before the initiating on CAT II treatment]
2. What is your understanding of presumptive MDR-TB criteria?
3. How is PMDT (from identification of presumptive to referral to diagnosis and treatment initiation) implemented in Bhopal? How is a patient with presumptive MDR-TB referred for DST? (does he go on his/her own or the sample is sent?)

*Interviewer will use the figure to describe the diagnosis and treatment pathway for presumptive /confirmed MDR-TB patients along with attrition at various levels. Problem is at the level of identification / referral*

1. PMDT should be screening all eligible presumptive MDR-TB patients. Why are all eligible presumptive MDR-TB patients not being detected? [Probe: programmatic, patient-level]
2. If detected, why are all presumptive MDR-TB patients not being referred for culture and DST? [Probe: programmatic, patient-level] [Probe: Major gap in identify/referral of patients; Probe – After reaching NRL – no attrition, minimal TAT]
3. How is the line listing of presumptive MDR-TB in the district made? [Probe: There is a presumptive MDR-TB / referral for CDST register at district level]
4. What are the operational issues involved in identification and referral of presumptive MDR-TB?: both at provider level and patient level? [Probe: do not know the criteria; know the criteria but no support for LT/TH-HV to courier the sample; rural/urban; other operational issues]
5. How can we further improve the identification/referral of presumptive MDR-TB cases?
6. Additional remarks, if any?

*Interviewer will complete the interview by acknowledging the time spared by the participant from his/her busy schedule. He will also give a summary of the notes taken and confirm the same from the participant.*
